# Supplementary material for: Ultrafast water sensing and thermal imaging by a metal-organic framework with switchable luminescence
Source: Nat Commun. 2017 Jun 30;8:15985. doi: 10.1038/ncomms15985 (PMC5497063; doi:10.1038/ncomms15985)
Supplement: Supplementary Information [file ncomms15985-s1.pdf]

File Name: Supplementary Information
Description: Supplementary Figures, Supplementary Tables, Supplementary Methods and Supplementary References

File Name: Supplementary Movie 1
Description: Emission colour change by N2 blow on paper-based film of LIFM–CL1-H2O (under 365 UV, direction 1）.

File Name: Supplementary Movie 2
Description: Emission colour change by N2 blow on paper-based film of LIFM–CL1-H2O (under 365 UV, direction 2).

File Name: Supplementary Movie 3
Description: Emission colour change by N2 blow on ZnO-supported film of LIFM–CL1-H2O (under 365 UV).

File Name: Peer Review File
Description:

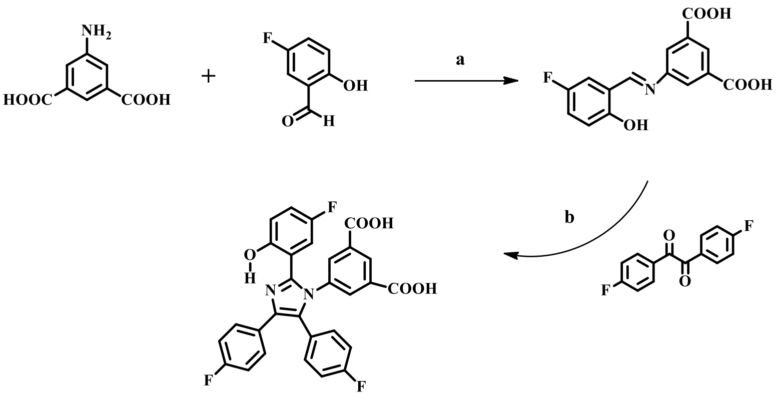

Supplementary Figure 1 | Synthetic route of ligand H_2hpi2cf. Reagents and conditions: (**a**) acetic acid, N_2 atmosphere, 110 °C, 2 h, 80%; (**b**) acetic acid, Diphenylethanedione, ammonium acetate, 110 °C, 10 h, 67%.

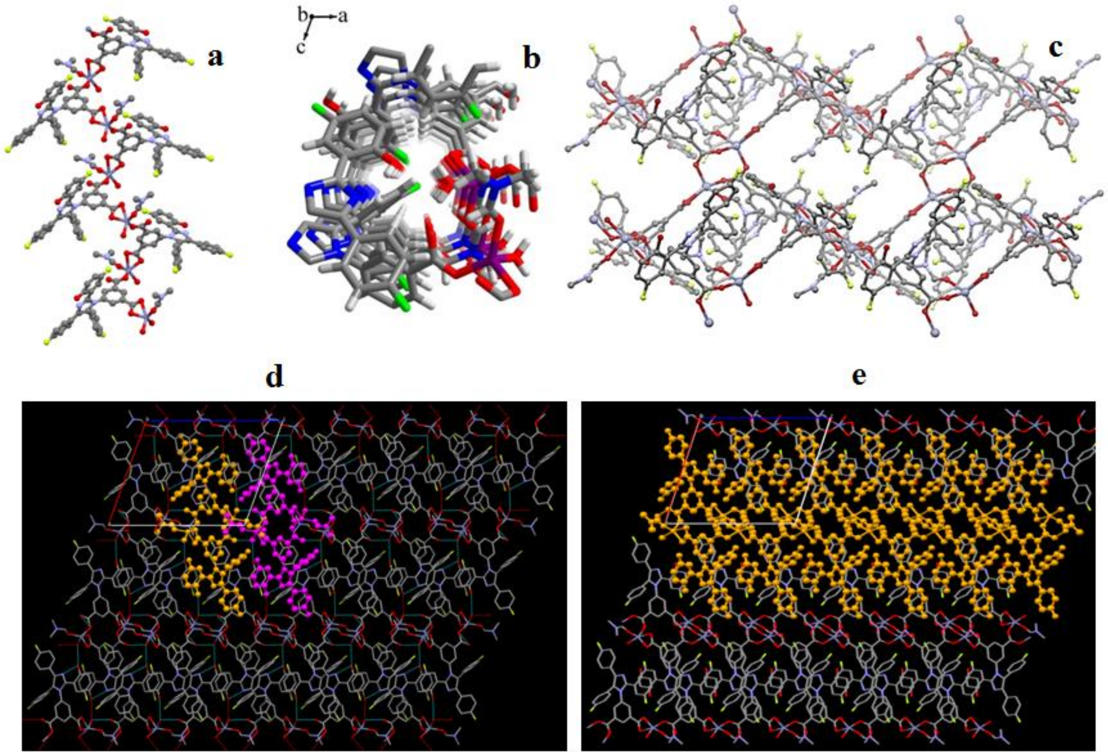

Supplementary Figure 2 | (**a**) One-dimensional chains in LIFM-CL1-H_2O crystal, (**b**) micropore along *b*-axis in LIFM-CL1-H_2O crystal, (**c**) 2D layer formed in dehydrated LIFM-CL1 via structural transformation mediated by coordinating-water removal and carboxylate O-Zn bond formation, (**d**) side-by-side packing allignment of 1D chains in LIFM-CL1-H_2O crystal lattice, forming H-bonds and π-stacking between chains, and (**e**) packing of 2D layers on *bc*-plane in LIFM-CL1 crystal.

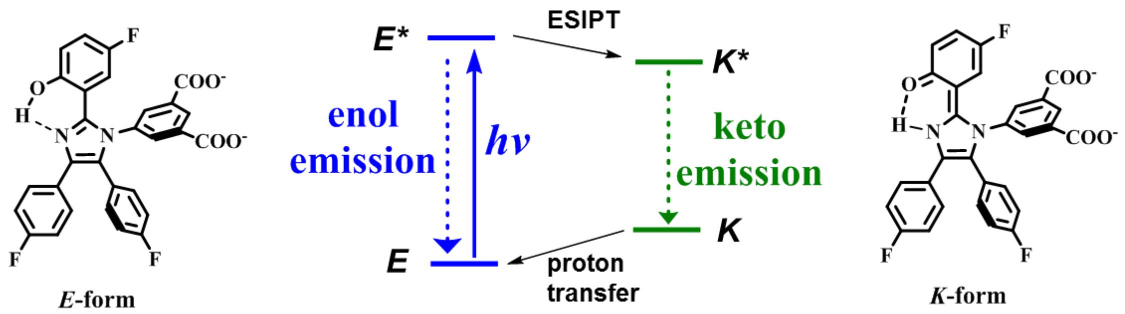

Supplementary Figure 3 | Chemical structure of ligand H_2hpi2cf in *E*-form and *K*-form, and emission levels relating to *E*-form and *K*-form of ligand based on ESIPT process. The tautomerization between *E*-form and *K*-form upon excitation is blocked in LIFM-CL1-H_2O by H-bonding but tuned-on in LIFM-CL1.

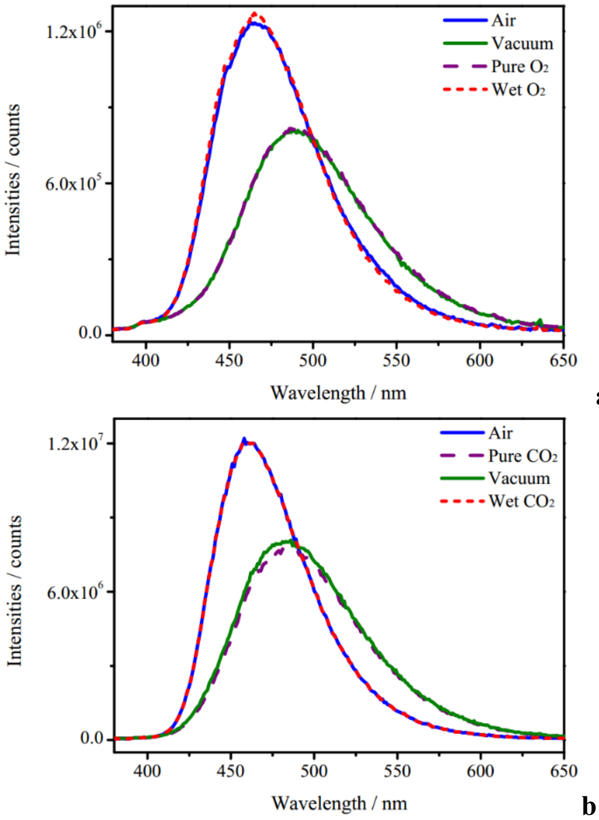

a

b

Supplementary Figure 4 | The comparison of emission spectra of LIFM-CL1-H_2O measured sequentially in air atmosphere (RH = 45% at 25 °C), vacuum, and *in-situ* gas flow of pure O_2 (**a**), CO_2 (**b**) and wet O_2 (**a**), CO_2 (**b**) containing water vapor that was collected from a bubbling tube filled with water.

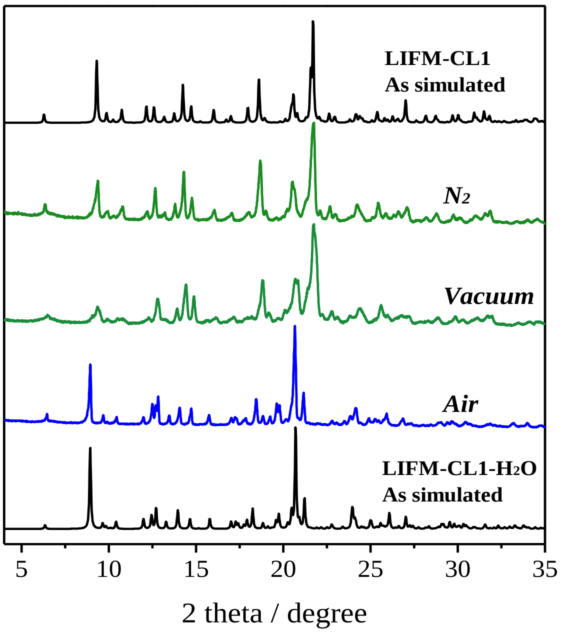

a

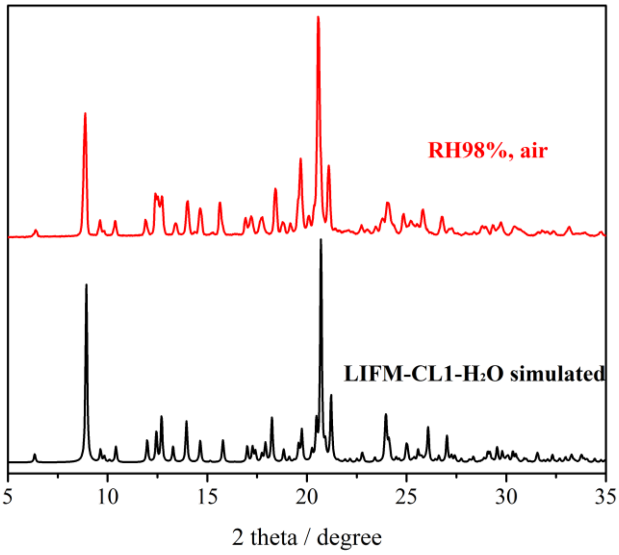

b

Supplementary Figure 5 | (**a**) The *in-situ* PXRD patterns of LIFM-CL1-H_2O measured in turn in ambient air, vacuum, and N_2 atmosphere, compared with the respective simulated XRD patterns; (**b**) the PXRD pattern of LIFM-CL1-H_2O measured in ambient air (RH 98%) compared with that of simulated pattern.

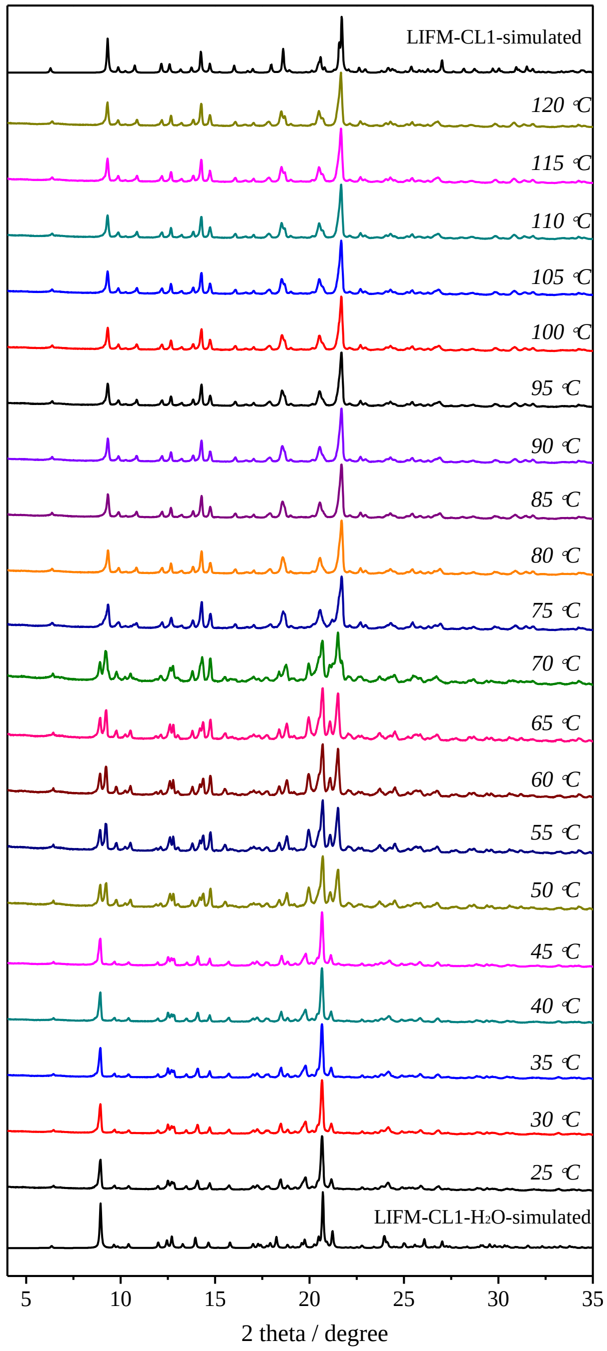

Supplementary Figure 6 | *In-situ* variable temperature PXRD patterns of Zn-MOF showing the transformation from LIFM-L1-H_2O to LIFM–CL1. The measurement was performed in air with the temperature rising rate of 1 °C min^{-1}, and each temperature was stablized for 5 minutes before the corresponding powder diffraction test.

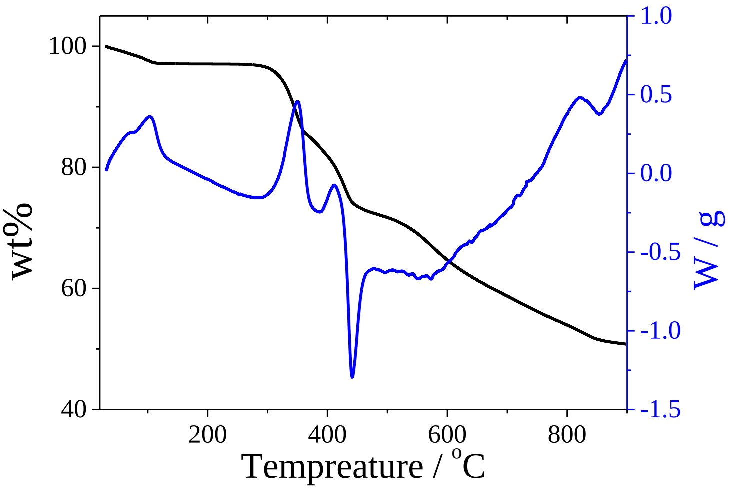

a

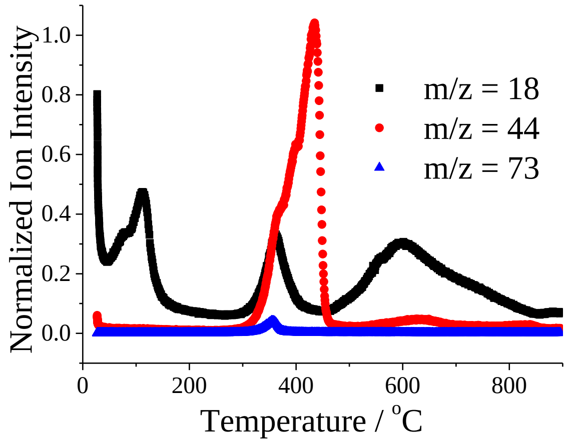

b

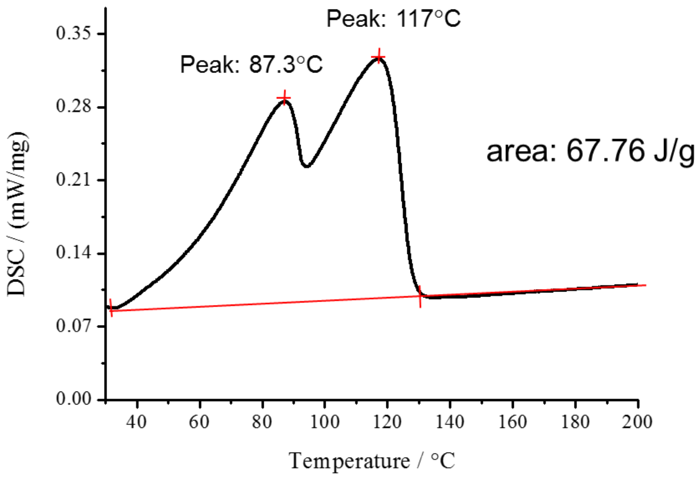

c

Supplementary Figure 7| TG, TG-MS and DSC of LIFM–CL1-H_2O. (**a**) TG shows a weight losses between 50-112 °C, corresponding to removal of coordinating water molecules. (**b**) TG-MS shows escape of water, CO_2 and DMF molecules in different heating stage. (**c**) DSC shows enthalpic change during water escape.

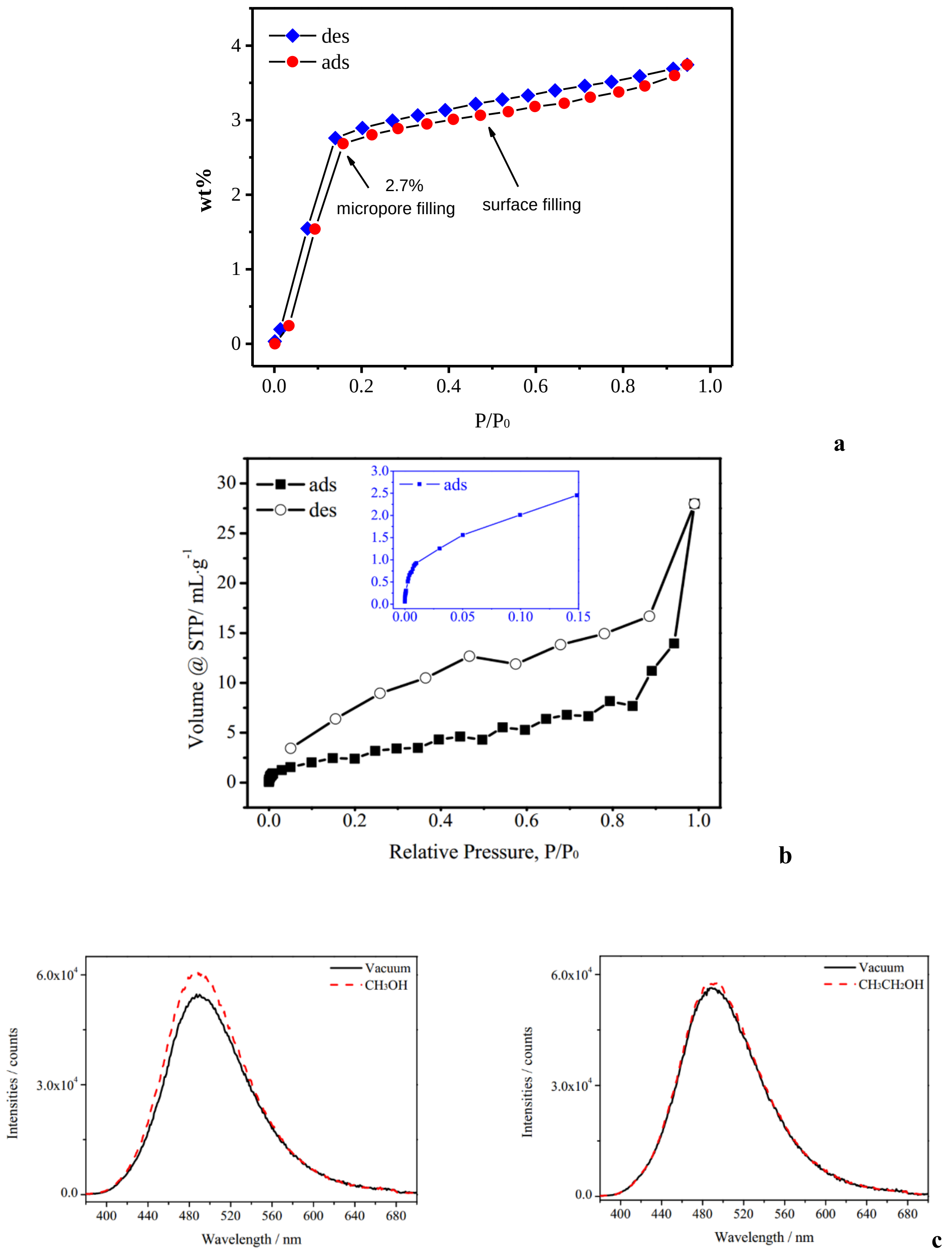

Supplementary Figure 8 | Water (**a**) and nitrogen **b**) adsorption and desorption isotherms at 298 and 77 K, respectively, for dehydrated sample LIFM-CL1, and the comparison of emission spectra (**c**) of dehydrated LIFM-CL1 sample measured in methanol and ethanol vapors. The dehydrated LIFM-CL1 was obtained by vacuumizing the hydrated LIFM-CL1-H_2O sample, and the alcohol vapors were generated from a N_2 bubbling tube filled with dry methanol or ethanol solvents.

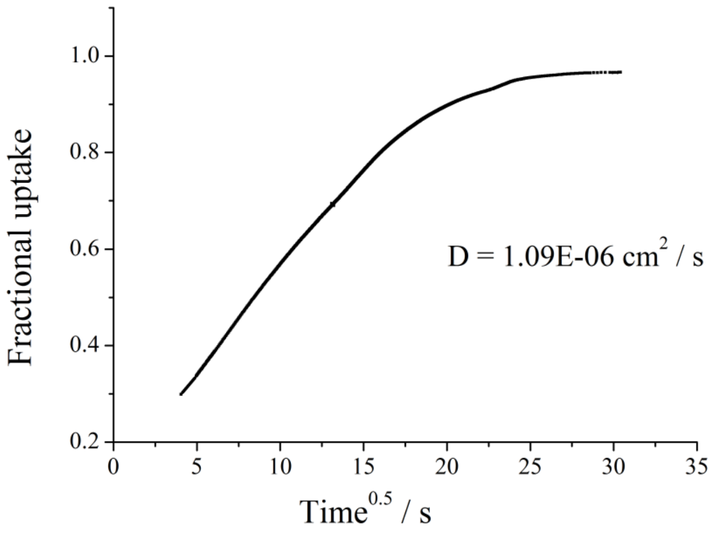

Supplementary Figure 9 | Kinetic profile of water vapor adsorption of LIFM–CL1 at 298 K.

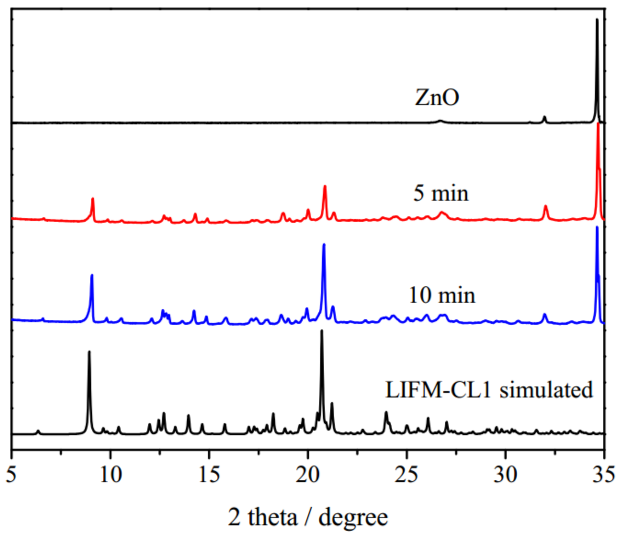

Supplementary Figure 10 | PXRD patterns of the *in-situ* grown LIFM-CL1-H_2O film at different times on the plated ZnO-precursor film. The peak of 26.4° is assigned to the FTO, and peaks at 32.1° and 34.8° are assigned to ZnO.

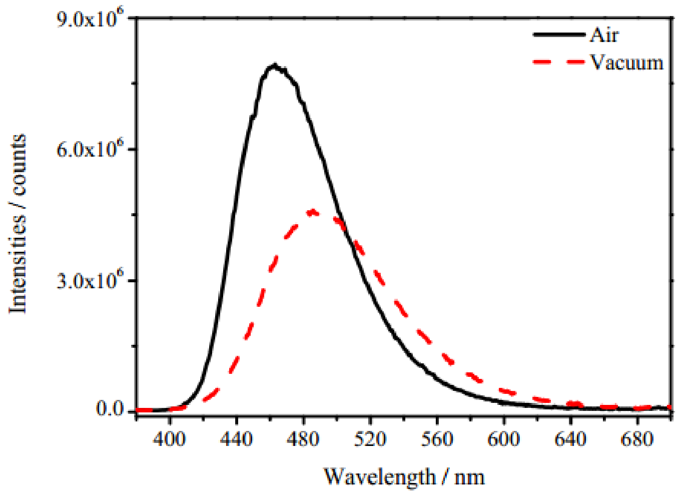

Supplementary Figure 11 | Emission spectra of the LIFM-CL1-H_2O-ZnO hybride film in air and vacuum (under 365 nm UV, 25°C)

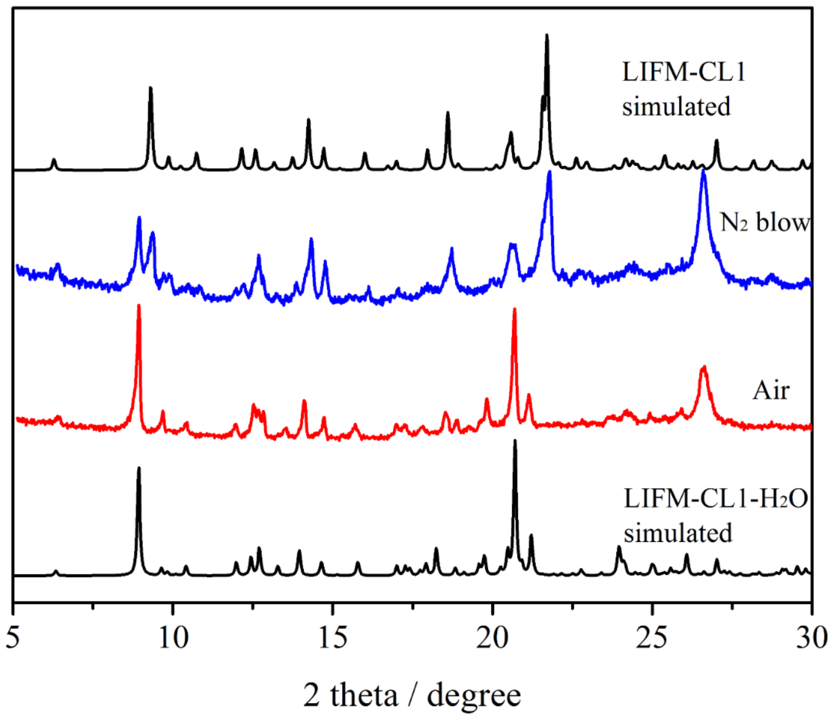

Supplementary Figure 12 | The corresponding XRD patterns of LIFM-CL1-H_2O-ZnO hybride film under N_2 blowing. A polyethylene tubule orifice was set on the film for N_2 blowing and the XRD pattern was collected simutaneously (10° min^{-1}). The peak of 26.4° was assigned to the FTO. As the nozzle we used for N_2 flow is very thin, so not the entire surface of LIFM-CL1-H_2O-ZnO hybrid film could be blown and led to structural transformation. Therefore the part of the film not blown by N_2 flow would keep the LIFM-CL1-H_2O phase, while this part was also exposed to X-ray beam and the PXRD pattern was collected together. And finally the PXRD pattern after N_2 blowing showed a mixture of the two forms.

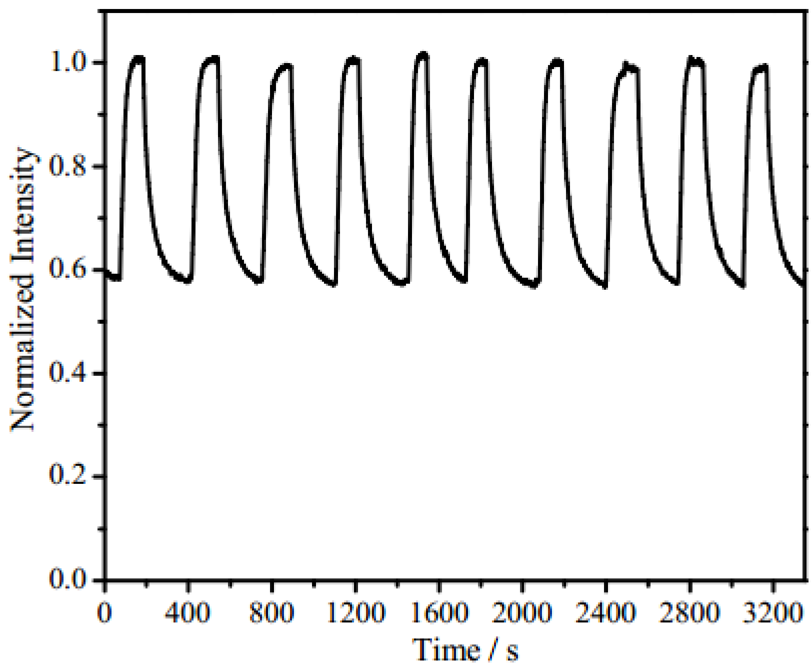

Supplementary Figure 13 | Time-dependent photoluminescence intensity switch (excited at 365 nm and detected at 463 nm) of the LIFM-CL1-H_2O-ZnO hybride film, which was cycled between vacuum and 1 bar air (RH = 45% on the testing day) at 25 °C.

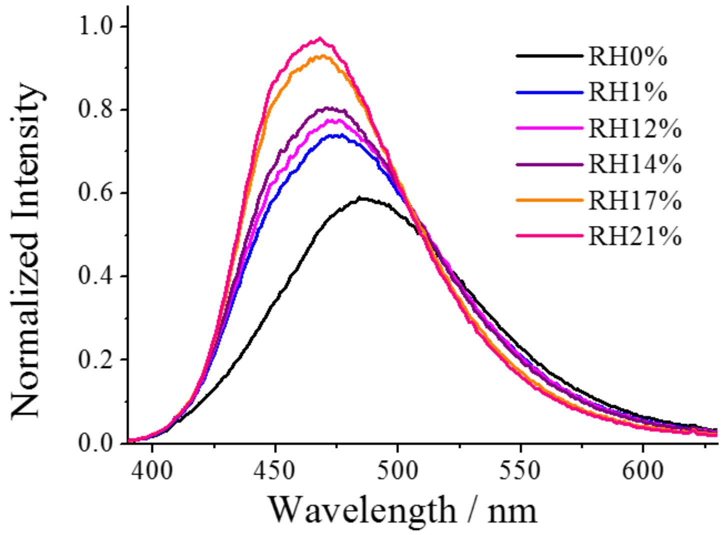

Supplementary Figure 14 | The comparison of emission spectra of dehydrated LIFM-CL1 film in N_2 atmosphere with different humidities generated by purging N_2 through H_2SO_4 containing different content of water and *in-situ* monitored by hygrometer.

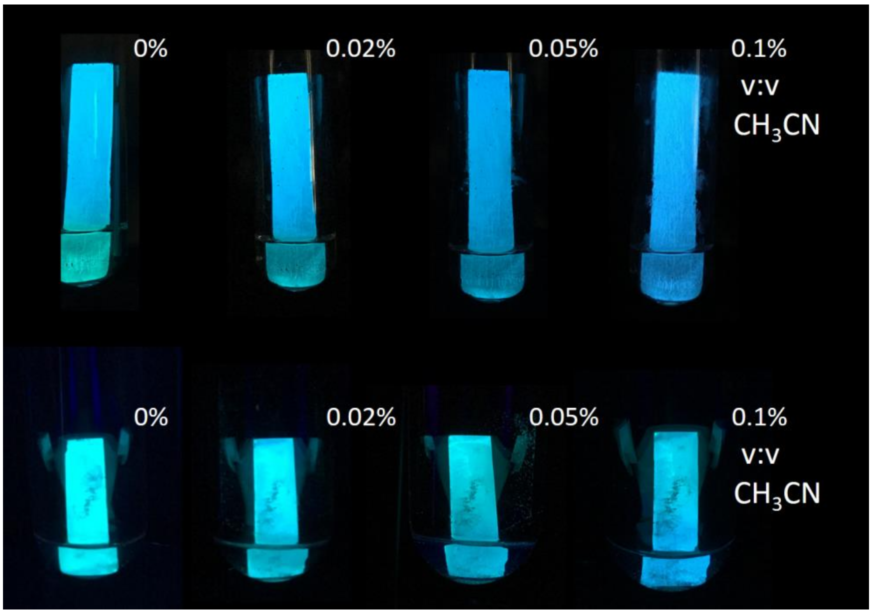

Supplementary Figure 15 | Illustration of the application of Zn-MOFs films as PL sensors detection of traces of water in organic solvents (excited at 365 nm). Photographs showing PL color change by immersing hydarted LIFM-CL1-H_2O-ZnO film or dehydrated LIFM–CL1–ZnO film partly into MeCN solutions containing different amounts of water (0, 0.02, 0.05, 0.1% v/v).

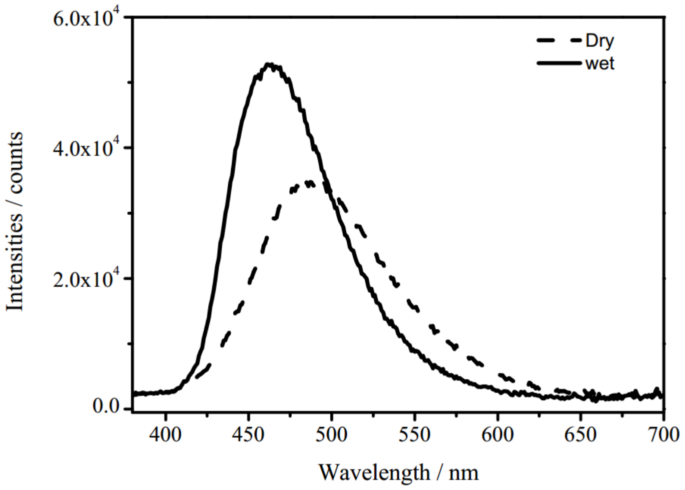

Supplementary Figure 16 | Emission spectra of the *in-situ* grown LIFM-CL1-H_2O-ZnO film in dry THF (dash) and wet THF (solid) with the water concentration of 0.05% , v/v (under 365 nm UV, 25°C).

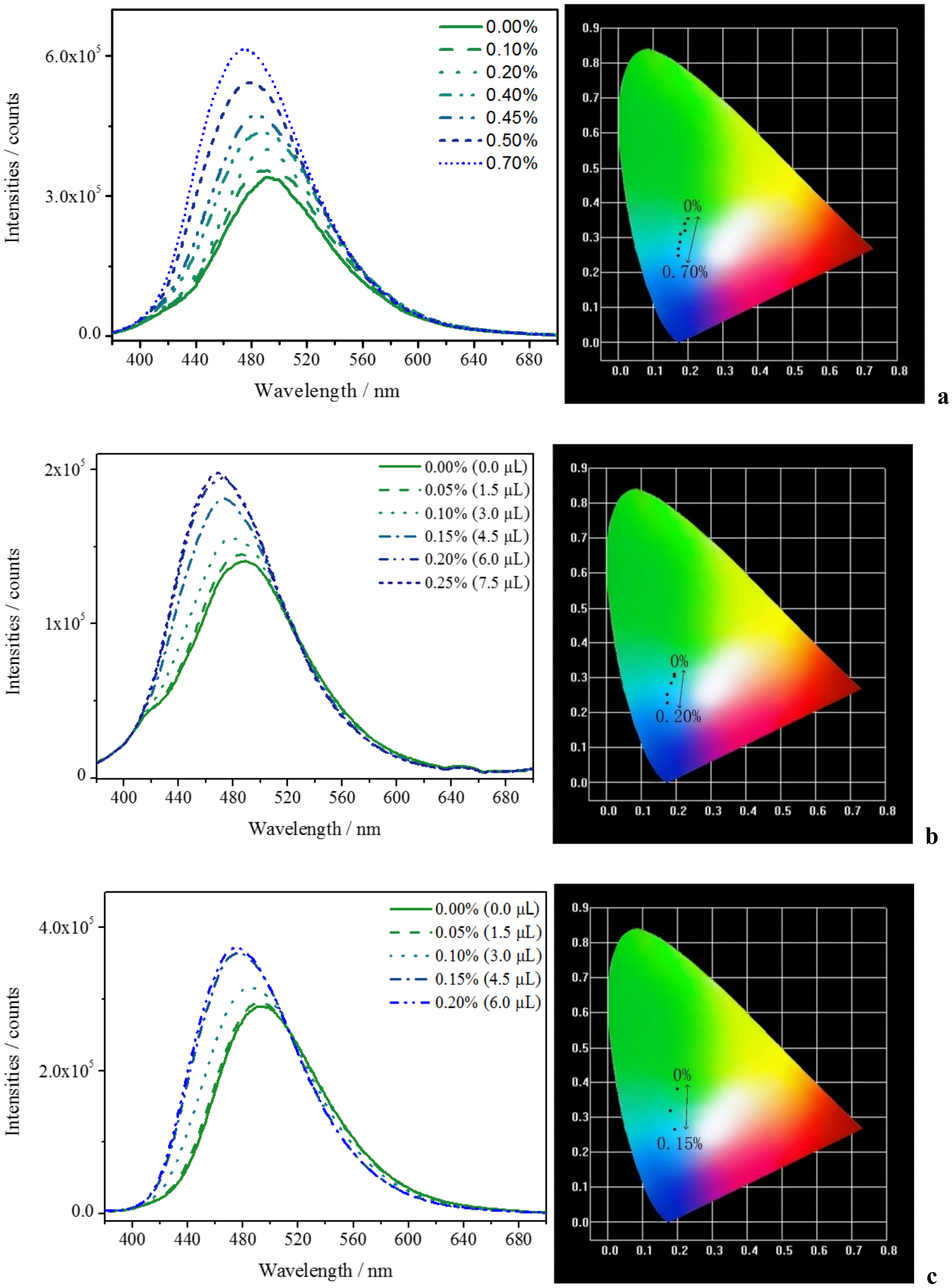

Intensities / counts
Wavelength / nm
0.00%
0.10%
0.20%
0.40%
0.45%
0.50%
0.70%
0%
0. 70%
a
0.00% (0.0 μL)
0.05% (1.5 μL)
0.10% (3.0 μL)
0.15% (4.5 μL)
0.20% (6.0 μL)
0.25% (7.5 μL)
0%
0. 20%
b
0.00% (0.0 μL)
0.05% (1.5 μL)
0.10% (3.0 μL)
0.15% (4.5 μL)
0.20% (6.0 μL)
0%
0. 15%
c

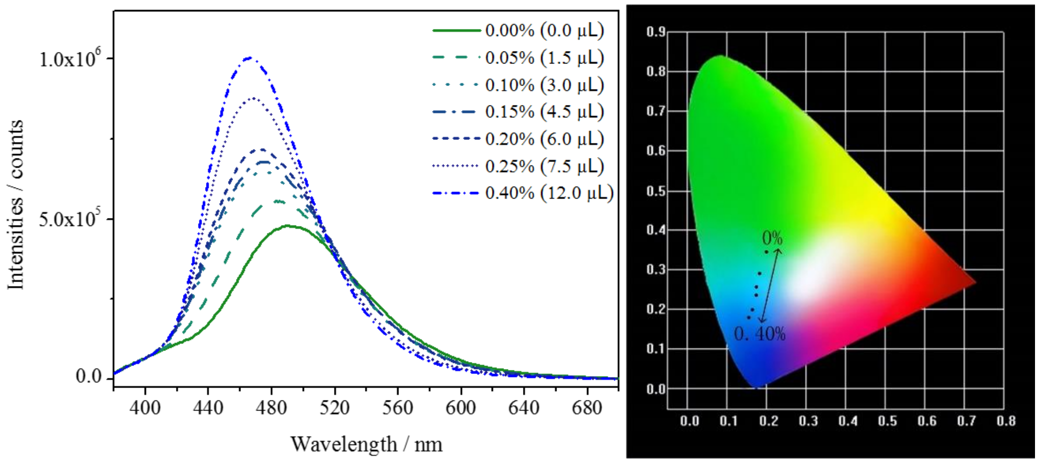

d

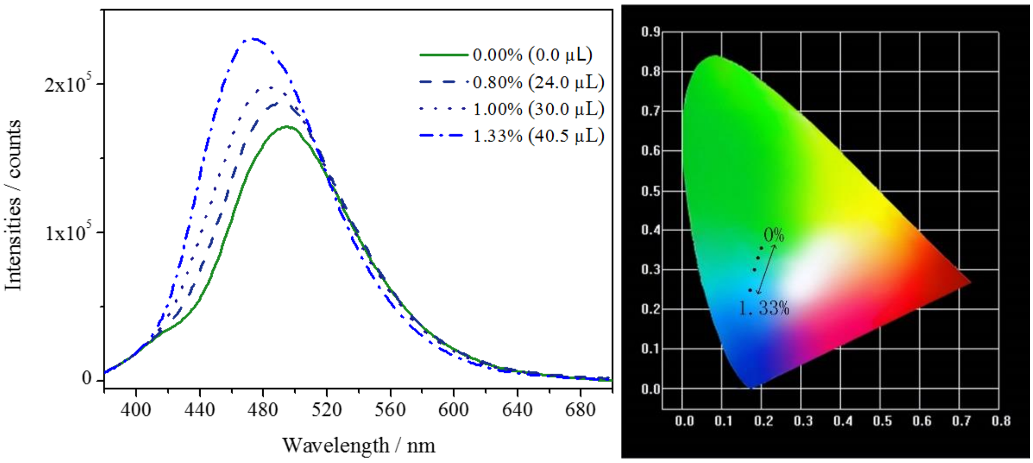

e

Supplementary Figure 17 | Changes in the emission spectra of a stirred suspension of dehydrated LIFM–CL1 microcrystals in dry organic solvents upon addition of aliquots of water (%v:v), and corresponding CIE coordinates of emission color. (**a**) ethanol (EtOH); (**b**) acetone; (**c**) acetonitrile (CH_3CN); (**d**) tetrahydrofuran (THF); (**e**) N,N-dimethylformamide (DMF). The excitation wavelength is 365 nm.

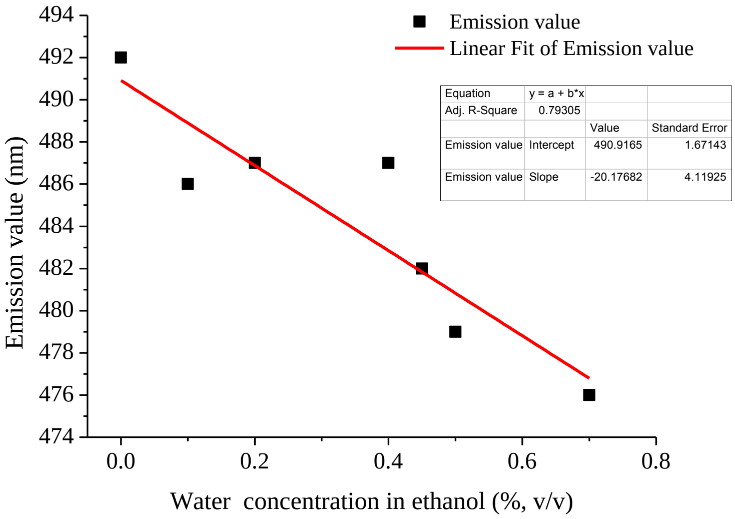

a

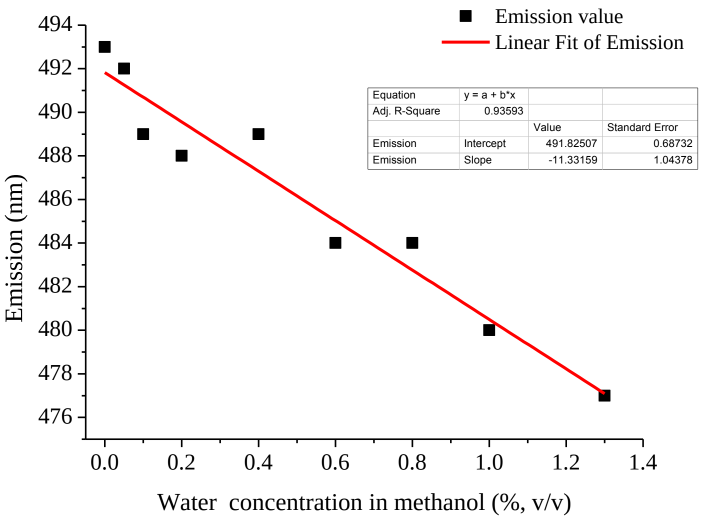

b

Supplementary Figure 18 | Linear relationship between water concentration vs. emitting wavelength of LIFM–CL1-H_2O powder sample suspended in EtOH (**a**) and MeOH (**b**).

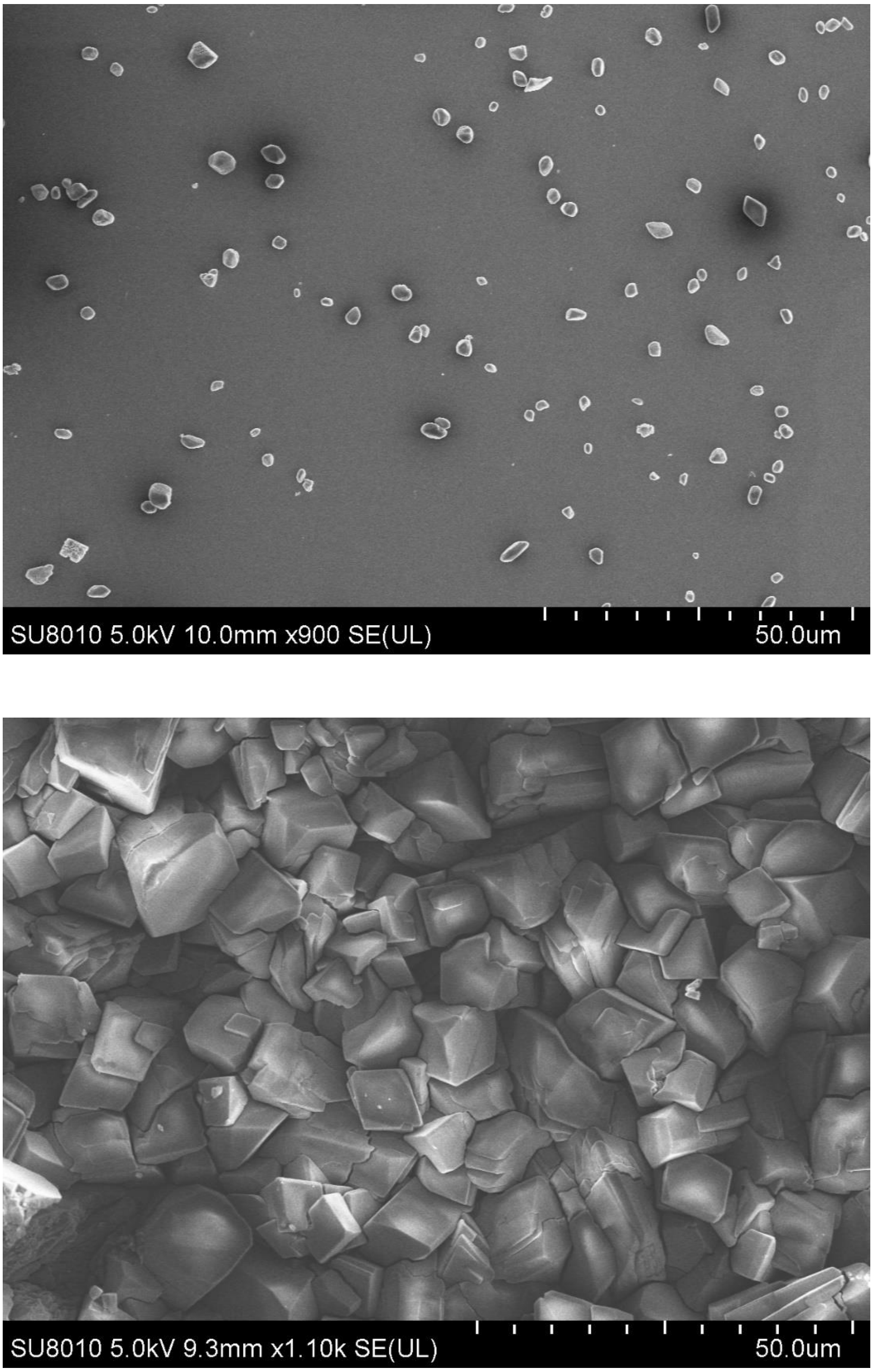

a

b

Supplementary Figure 19 | SEM images of LIFM-CL1-H_2O (**a**) microcrystallines synthesized by stiring hydrothermal method and (**b**) synthesized by *in-situ* growing method onto the ZnO-nanorod film.

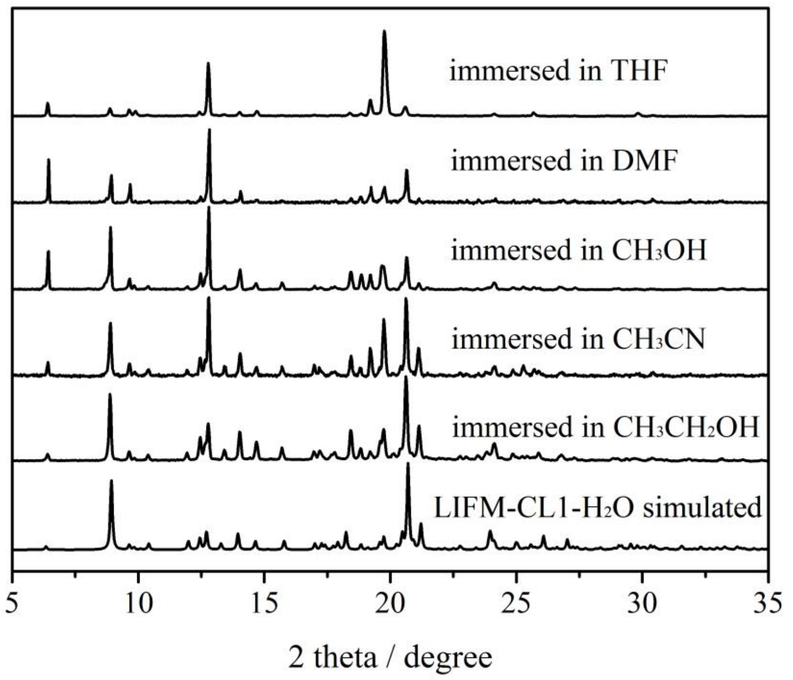

Supplementary Figure 20 | PXRD patterns of LIFM-CL1-H_2O powder (ground sample from crystals) immersed in organic solvents for two hours.

Supplementary Table 1│Crystallographic data and structure refinement for LIFM–CL1 and LIFM–CL1-H_2O (the single-crystal X-ray diffraction data are collected on the same crystal before and after N_2 blow at 297 K, and at 358 K).

Cell parameters	Crystal LIFM-CL1-H2O	Crystal LIFM-CL1-H2O	Crystal LIFM-CL1	Crystal LIFM-CL1
Formula	$ZnC_{32}H_{24}F_3N_3O_7$	$ZnC_{32}H_{24}F_3N_3O_7$	$ZnC_{32}H_{22}F_3N_3O_6$	$ZnC_{32}H_{22}F_3N_3O_6$
Temperature (K)	150	297	297, N_2 blow	358
Wavelength (Å)	Cu Kα, 1.5418	Cu Kα, 1.5418	Cu Kα, 1.5418	Cu Kα, 1.5418
Crystal system	Monoclinic	Monoclinic	Monoclinic	Monoclinic
Space group	*P2₁/c*	*P2₁/c*	*P2₁/c*	*P2₁/c*
Unit cell dimensions	a = 14.9914(2) Å	a = 14.7944(2) Å	a = 14.7953(4) Å	a = 14.8070(4) Å
	b = 12.0248(1) Å	b= 12.2949(2) Å	b = 11.6024(3) Å	b = 11.7383(4) Å
	c = 18.1645(3) Å	c = 18.1623(3) Å	c = 17.3581(5) Å	c = 17.3054(6) Å
	α= 90 °	α = 90 °	α = 90 °	α = 90 °
	β = 111.468(2) °	β = 110.117(2) °	β = 108.351(3) °	β = 108.442(3) °
	γ = 90 °	γ = 90 °	γ = 90 °	γ = 90 °
Volume ($Å^3$)	3047.31(8)	3102.09(9)	2828.18	2853.37(17)
Z	4	4	4	4
Dcalc ($g·cm^{-3}$)	1.493	1.467	1.566	1.552
Mu (mm^{-1})	0.877	0.862	0.941	0.932
F(000)	1400	1400	1360.0	1360.0
GOF	1.079	1.040	1.038	1.062
R1(all)	0.0520(4910)	0.0732(5218)	0.0555(4624)	0.0619(4773)
wR2 [I>2σ(I)]	0.1616(5457)	0.1980(6075)	0.1709(5534)	0.1861(5555)

Supplementary Table 2│Photophysical properties of complex LIFM-CL1 and LIFM-CL1-H_2O.

**Form**	**Em**, $\lambda_{/nm}$	**PLQY**/%	**Lifetime**, $\tau_{/ns}$
LIFM-CL1	463	22	5.21
LIFM-CL1-H_2O	493	15	5.52

**Supplementary Table 3 | **Comparison of key parameters of reported fast luminescence water sensors.

Luminophor	Phase as sensing material	Chemical formula	Emission position shift / nm	Intensity change	Water content	Activation/ Recovery	Interference molecule	Responding time	Ref
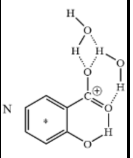	Mixture: doped poly(vinyl-alcohol) (PVA) film	$C_7H_9NO_5^+$	--[a]	Decreased	RH from 5% to 85%; Concentration 0% -60% v/v in 1,4-dioxane	< 1 minute, As PVA desorbs H_2O quickly	--[b]	2 minutes	1
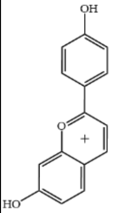	Mixture: doped poly(2-hydroxyethyl methacrylate) (PHEMA) polymer	$C_{15}H_{11}0_3^+$	--[a]	Decreased	RH from 20% to 100%	Dried overnight in a desiccator containing silica gel	NH_3	--[b]	2
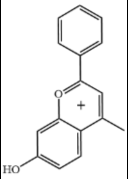	Mixture: doped poly(2-hydroxyethyl methacrylate) (PHEMA) polymer	$C_{16}H_{13}O_2^+$	--[a]	Decreased	RH of 80%	Dried overnight in a desiccator containing silica gel	NH_3	1 hour	
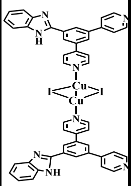	2D net	$C_{24.50}H_{20}CuIN_{4.50}O_{1.25}$	607 to 613	Increased	RH of 33%, 43%, 57%, and 75.8%.	guest-exchange	Depending on guest species encapsulation	1.5 to 4 hours	3
Eu^{3+} or Tb^{3+} as counter cation in a 3D porous framework		$C_{11}H_{11}O_9Ln$	No shift	Increased	RH of 5%, 30%, 48%, 75.8% and 85%.	heating at 200 °C for 2 h	Not mentioned	1 to 24 hours	4
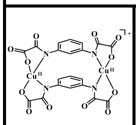	3-connected 2D net	$C_{72}H_{38}N_{14}O_{36}Cu_9Mn_2$	586 to 544	Increased	Concentration 0-33 wt% in toluene.	vacuum and heating at 373 K for 16 h	MeOH	More than 60 minutes	5
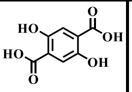	Connected 3D irl net	$C_8H_8O_8Mg$	455 to 530	Increased	Concentration 0.05 - 5% v/v in organic solvents.	MeOH-exchange plus gentle heating at 50 °C	MeOH, EtOH, CH_3CN, DMAc	2 minutes	6

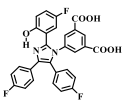	3D structure connected by 1D chains via H-bonding and π stacking	$C_{32}H_{22}F_3N_3O_6$	493 to 463	Increased	RH < 1%; Concentration < 0.05% v/v in organic solvents; Quantitative 0-1.3% v:v	dry gas blowing or heating at 70 °C	Only responsive to water	For gas: ~2 seconds. For liquid: instantly.	This work

[a] No emission position shift. [b] Not mentioned.

Supplementary Table 4 | Examination of water content of the used CH_3OH solvents by Karl Fischer Coulometric Titrimetry.

Theoretical value	V (H_2O) / 3 mL CH_3OH	0	1.5 μL	3.0 μL	6.0 μL
	V / V	0%	0.05%	0.10%	0.20%
Measured value	Detected/ ppm (mg/kg)	316.4	957.3	1539.3	2866.1
	V / V	0.0251%	0.0758%	0.1219 %	0.2271 %

Supplementary Table 5 | Confirmation of water content of CH_3OH solvents before and after being soaked with hydrated LIFM-CL1-H_2O and dehydrated LIFM-CL1 by Karl Fischer Coulometric Titrimetry.

Mass of sample (mg)			hydrated	dehydrated
			3.1	2.2
Water content (v/v)	before	Measured/ppm (mg/kg)	316.4	2866.1
		V / V	0.0251%	0.2271 %
	after	Measured/ppm (mg/kg)	412.1	2718.5
		V / V	0.0326%	0.2154%

Supplementary Methods

Synthesis of H_2hpi2cf

A mixture of 5-aminoisophthalic acid (5 mmol, 0.905 g) and 5-fluoro-2-hydroxybenzaldehyde (3 mmol, 0.420 g), acetic acid (20 mL) were stirred at 110°C for two hours under the protection of nitrogen, continued with adding 4, 4'-difluorobenzil (3 mmol, 0.738 g), ammonium acetate (50 mmol, 3.85 g), then the mixture were kept heated and stirred for another 10 hours. After cooling, the white solid was filtered out, washed by water for several times and then dried at 100 °C in air. Recrystallization in the ethyl acetate affords colorless crystal product (530 mg, yield = 67%).[7] Compound H_2hpi2cf was analyzed as H_2hpi2cf·DMF·EtOH. Anal calcd. for $C_{34}H_{30}F_3N_3O_7$ (%): C, 62.86; H, 4.65; N, 6.47; found: C, 62.68; H, 4.563; N, 6.72. IR (KBr pellet, cm^{-1}): ν = 3471 (-O-H), 3075 (Ar-H), 1705 (-C=O), 1630 (-C=N-), 1510 (C=C), 670, 780 (C-H). ^{1}H NMR (400 MHz, DMSO-d_6, δ): 8.34 (t, J = 1.4 Hz, 1H, carboxyl-H), 7.95 (s, 1H, 1-Ar-H), 7.90 (d, J = 1.5 Hz, 2H, 1-Ar-H), 7.53 – 7.42 (m, 2H, Ar-H), 7.36 – 7.27 (m, 2H, Ar-H), 7.17 (dt, J = 16.1, 8.9 Hz, 4H, Ar-H), 7.06 (td, J = 8.6, 3.2 Hz, 1H, 2-Ar-H), 6.93 (dd, J = 9.4, 3.1 Hz, 1H, 2-Ar-H), 6.77 (dd, J = 9.0, 4.9 Hz, 1H, 2-Ar-H).

Supplementary References

(1) Mishra, H., Misra, V., Mehata, M. S., Pant, T. C. & Tripathi, H. B. Fluorescence studies of salicylic acid doped poly(vinyl alcohol) film as a water/humidity sensor. *J. Phys. Chem. A* **108**, 2346-2352 (2004).

(2) Galindo, F. *et al*. Water/humidity and ammonia sensor, based on a polymer hydrogel matrix containing a fluorescent flavylium compound. *J. Mater. Chem.* **15**, 2840-2847 (2005).

(3) Yu, Y. *et al*. Cu(I)-MOF: naked-eye colorimetric sensor for humidity and formaldehyde in single-crystal-to-single- crystal fashion. *Chem. Commun.* **50**, 1444-1446 (2014).

(4) Yu, Y., Ma, J.-P. & Dong, Y.-B. Luminescent humidity sensors based on porous Ln^{3+}-MOFs. *CrystEngComm* **14**, 7157-7160 (2012).

(5) Ferrando-Soria, J. *et al*. Highly selective chemical sensing in a luminescent nanoporous magnet. *Adv. Mater.* **24**, 5625-5629 (2012).

(6) Douvali, A. *et al*. Turn-on luminescence sensing and real-time detection of traces of water in organic solvents by a flexible metal–organic framework. *Angew. Chem. Int. Ed.* **54**, 1651-1656 (2015).

(7) Chen, L. *et al*. A naked eye colorimetric sensor for alcohol vapor discrimination and amplified spontaneous emission (ASE) from a highly fluorescent excited-state intramolecular proton transfer (ESIPT) molecule. *J. Mater. Chem. C* **4**, 6962-6966 (2016).
